# Enabling chlorophyll photo-response for in-line real-time noninvasive direct probing of the quality of palm-oil during mill process

**DOI:** 10.1038/s41598-023-32479-7

**Published:** 2023-04-07

**Authors:** Eddie Khay Ming Tan, Soon Huat Tiong, Dalina Adan, Mohd Zairey bin Md. Zain, Syahril Anuar Md Rejab, Mohd. Shafril Baharudin, Hao Chih Loy, Eng Soon Tok, Wee Lee Tok, David Ross Appleton, Huey Fang Teh

**Affiliations:** 1 TechnoSpex Pte. Ltd., 1092 Lower Delta Road, #04-01 Tiong Bahru Industrial Estate, Singapore, 169203 Singapore; 2 Sime Darby Plantation Technology Centre Sdn. Bhd., UPM-MTDC Technology Centre III, 1st Floor, Block B, Lebuh Silikon, 43400 Serdang, Selangor Darul Ehsan Malaysia; 3 Sime Darby Research Sdn. Bhd., Lot 2664, Jalan Pulau Carey, 42960 Pulau Carey, Selangor Darul Ehsan Malaysia; 4 RGS Corporation Sdn. Bhd. Serdang Sky Villas, Lot SB 15, Jalan SP5/5, Taman Serdang Perdana, 43300 Seri Kembangan, Selangor Darul Ehsan Malaysia; 5 ɛMaGIC-Lab, Department of Physics, National University of Singapore, Singapore, 117551 Singapore; 6 Present Address: Home Team Science and Technology Agency (HTX), 1 Star Avenue, #12-01, Singapore, 138507 Singapore

**Keywords:** Chemistry, Optics and photonics, Physics

## Abstract

During the milling process of palm oil, the degree of palm fruit ripeness is a critical factor that affects the quality and quantity of the oil. As the palm fruit matures, its chlorophyll level decreases, and since chlorophyll in oil has undesirable effects on hydrogenation, bleachability, and oxidative degradation, it’s important to monitor the chlorophyll content in palm oil during the milling process. This study investigated the use of light-induced chlorophyll fluorescence (LICF) for non-invasive and real-time monitoring of chlorophyll content in diluted crude palm oil (DCO) located at the dilution and oil classification point in palm oil mill. An LICF probe was installed at the secondary pipe connected to main DCO pipeline, and the system communicates with a computer located in a separate control room via a Wi-Fi connection. Continuous measurements were recorded with an integration time of 500 ms, averaging of 10, and a time interval of 1 min between each recording during the oil mill’s operation. All data were stored on the computer and in the cloud. We collected 60 DCO samples and sent them to the laboratory for American Oil Chemists’ Society (AOCS) measurement to compare with the LICF signal. The LICF method achieved a correlation coefficient of 0.88 with the AOCS measurements, and it also provided a direct, quantitative, and unbiased assessment of the fruit ripeness in the mill. By incorporating Internet of Things (IoT) sensors and cloud storage, this LICF system enables remote and real-time access to data for chemometrics analysis.

## Introduction

Palm oil is an edible oil that derived from the fruit of the oil palm, *Elaeis guineensis*. *Tenera*, derived from a cross between Deli *Dura* and AVROS *Pisifera* palms, is primarily bred in Southeast Asia for production^1^. It is known that oil palm fruits of different ripeness levels have varying chemical compositions, particularly in term of oil content. For instance, the content of neutral lipid, in oil palm mesocarp, increases rapidly 16 weeks after pollination (WAP) and it reaches a maximum typically at 20 WAP^2–4^. Therefore, it is critical in the production process flow that the fresh fruit bunches (FFB) are harvested at optimal ripeness and also processed as soon as possible to produce good quality oil with an optimal oil extraction rate^5^. In general, when the harvested FFBs arrive at the oil mill, they can show different degree of ripeness ranging from under-ripe to unripe, ripe, over-ripe and rotten. The current practice of grading the FFB ripeness at the mill is also a laborious manual process. Ripeness assessment is done using human graders who count the number of loose fruit sockets on the bunch and access the shades of colours observed from the fruit. Depending on experience level of the assessors, inconsistent and inaccurate judgment can arise, which can affect the total and quality of the oil content extracted during mill operations.

To aid the process, technologies have been developed over years to determine the maturity level of FFB. These include computer vision (using red–green–blue imaging) integrated with laser-light backscattering techniques^6^, spectroscopy^7^, hyperspectral imaging^8^, thermal imaging^9^, inductive frequency technique^10^ and electrochemical sensor based on the fruit battery principle^11^. More recently, Raman^7,12,13^, Infrared^14^, NIR^15,16^, optical sensor^17^ or diffuse reflectance spectroscopy^18^ have also been used to classify oil palm fresh fruit maturity based on carotene and chlorophyll content. Carotene and chlorophyll are extracted along with palm oil during the extraction process from the palm fruit mesocarp. Interestingly, the level of chlorophyll content has an inverse relationship with fruit maturity. During the unripe stage (before WAP 17), chlorophyll content is highest and it decreases as the fruits ripen (WAP 18–22)^19,20^. The surface of the fruit is usually green in color during the unripe stage due to the high concentration of chlorophyll, and it turns orange-red in color during the ripening stage due to the accumulation of carotene and the degradation of chlorophyll^21^. During the early stage, the chlorophyll on the surface of the FFB is actively involved in photosynthesis, producing energy that is essential for the growth and development of the fruit. As the fruit matures, the role of chlorophyll becomes less crucial, and the fruit begins to accumulate phytonutrients and other compounds, such as carotene, which give it its final orange-red color. These color changes can be used to determine the maturity and ripeness of the fruit, which is important in the harvesting and processing of oil palm fruits. Hazir et al.^22^ used a hand-held multiparameter fluorescence sensor to manually classify the FFB based on their ripeness. The study showed that fluorescence spectroscopy combined with chemometric analysis was able to accurately classify oil palm FFB according to their ripeness with an accuracy of up to 89.7%. Bensaeed et al.^8^ conducted a study on the ripeness detection of oil palm fruits (*nigrescens*, *virescens*, and *oleifera*) using a hyperspectral sensor integrated with artificial neural network classification algorithms, and achieved an accuracy of more than 95% for all three types of oil palm fruits.

High chlorophyll content in crude palm oil would indicate the presence of oil extracted from unripe fruits. Furthermore, the presence of chlorophylls in oil is undesirable as chlorophylls are photosensitizers; hence their presence in the palm oil will lead to enhance photo-oxidation of the palm oil^21,23–26^. Chlorophyll fluorescence has widely been used in agriculture to monitor, the photosynthetic activities in plants non-destructively and to detect plant stress, among other^27–29^. Another interesting use of chlorophyll fluorescence is as a screening tool for fruit and vegetable maturity^30–32^. Abdelhamid et al. developed a LED-based light-induced chlorophyll sensor for monitoring tomato ripening stages^32^. They reported that as the level of chlorophyll fluorescence decreases with tomato maturity, indicating the reduction of chlorophyll. This result agrees with what has been observed in palm fruits^4^ and palm oil extracted from palm fruits with different levels of maturity^7,24^. Y. A. Tan et al. found that the measured chlorophyll fluorescence intensity is directly correlated to different amount of dissolved chlorophyll A added to refined palm kernel oil^33^. These alternative optical-based approaches suggest that by monitoring the chlorophyll content, it can be leveraged to estimate the FFB ripeness of palm fruit. As an optical probe, it also has the potential to be used in the production mill with minimum disruption.

DCO is crude palm oil produced in the mill after pressing of sterilized oil palm fruits, which are mixed with hot water to separate crude palm oil (CPO) from other components, such as debris, sand, and non-oily solids. However, monitoring chlorophyll in DCO is challenging due to the potential interference of these matrix presence in the DCO. Assessing the chlorophyll content at this early stage of the palm oil production would be useful as it can alert the production team to the presence of high chlorophyll content in the oil pipeline. However, the current analysis process is done off-line, and it requires an operator to station at the mill to periodically collect the DCO sample for chlorophyll measurements in the laboratory. The current method used to determine chlorophyll content in crude vegetable oils is performed following the American Oil Chemists’ Society (AOCS) Cc 13 k-13 method^34^. AOCS Cc 13 k-13 is a standard method for determining chlorophyll pigments in vegetable oils using spectrophotometry. The method involves measuring the absorbance of light at three different wavelengths (630 nm, 670 nm and 710 nm) and using the results to calculate the pheophytin, which is the main pigment in chlorophyll, content in the vegetable oil samples. The current practice is, therefore, a time-consuming, disruptive and labour-intensive process, which does not provide direct, real-time monitoring of the chlorophyll content level of the produced oil.

The aim of this work is to demonstrate that light-induced chlorophyll fluorescence (LICF) is a suitable technique to monitor the chlorophyll content in the DCO in an industrial production palm oil mill. This is the first prototype developed for real-time chlorophyll level monitoring in palm oil production mill. Ex-situ-laboratory-based measurements will first be described, and this is followed by building of the non-invasive prototype and the eventual implementation of the prototype in the actual palm oil production mill in Malaysia for in-line real-time monitoring. We will show that the results obtained correlate well with the AOCS data.

This paper consists of three main sections of experimental works. The first section is divided into two parts. In the first part of the work, chlorophyll fluorescence was measured using LICF to probe different chlorophyll concentration solutions. The chlorophyll solutions were prepared by dissolving different concentrations of chlorophyll A in methanol. This experiment provided a starting point to validate the feasibility of LIF for chlorophyll detection. In the second part, we measured the chlorophyll in the DCO extracted from fruits at different FFB maturity stages.

In the second section of the experiment, we conducted a feasibility study to measure chlorophyll in DCO samples collected directly from the mill without any further treatment in the laboratory. As mentioned, DCO contains not only crude palm oil but also other matrices such as fibers from the fruit and soil. The purpose of the study was to establish whether LICF is still a viable technique for measuring chlorophyll in DCO and whether the results are affected by the matrices present in the DCO. The measured results were correlated with the AOCS outcome. Note that all the experiments were carried out in the laboratory under controlled conditions.

The third section of the experimental work focuses on the development of the LICF prototype and its implementation in the palm oil mill for real-time, continuous monitoring of chlorophyll levels in the DCO flowing through the pipeline. During the monitoring of chlorophyll levels, samples were collected randomly for AOCS analysis, and the results were compared to the LICF data.

## Methods

### Chlorophyll analysis with LICF

To investigate the response of chlorophyll to 405 nm excitation light and the relationship between chlorophyll fluorescence intensity and chlorophyll concentration level, we measured a series of chlorophyll solutions at various controlled concentrations. Six chlorophyll solutions were prepared by dissolving different amount of chlorophyll A (Sigma Aldrich) in methanol. The different concentrations prepared were 100 µg/L, 80 µg/L, 60 µg/L, 40 µg/L, 20 µg/L, 10 µg/L and 5 µg/L. Each chlorophyll solution sample was first poured into a UV-plastic cuvette and illuminated with 405 nm LED light with a power output of 15mW at the distal end of the probe. The fluorescence signal was acquired with an integration time of 500 ms and averaging of 10.

### Chlorophyll analysis in DCO with AOCS method

Chlorophyll in DCO as produced in the mill was analyzed as-received using a modified AOCS method (Cc 13 k-13). Chlorophyll-related pigments (predominantly pheophytin a) were determined from spectrophotometric absorption measurements at 630, 670 and 710 nm. Prior to the measurement the DCO samples were heated to 60 °C, followed by centrifuging at 1000 rpm for 15 min. The aliquot of oil layer then undergoes the filtration process before transferred to 10 mm plastic cuvette. The content of chlorophyll is expressed in mg of pheophytin a, which is calculated as follows:

$$\begin{gathered} {\text{C}}\,{ = }\,\frac{{345.3\, \times \,\left[ {{\text{A}}_{670} \,{-}\,\left( {0.5\, \times \,{\text{A}}_{630} } \right)\,{-}\,\left( {0.5\, \times \,{\text{A}}_{710} } \right)} \right]}}{{\text{L}}}, \hfill \\ \hfill \\ \end{gathered}$$

where, C = content of chlorophyll pigments as mg of pheophytin a in 1 kg of oil, A = Absorbance at the respective wavelength (nm), L = light path of the spectrophotometer cell (mm).

### LICF setup for laboratory

**Figure 1:**
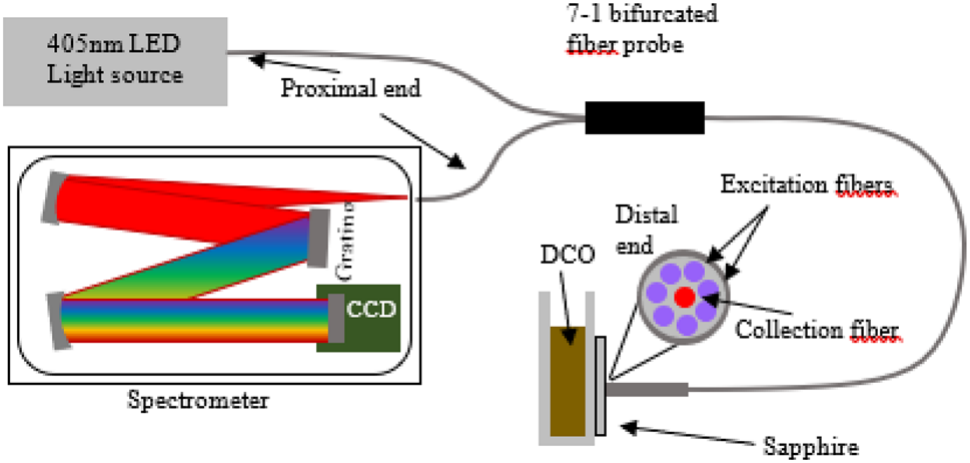
Schematic diagram of LICF setup used in the laboratory for the LIF experiment.

The LICF system optical setup consists of three sections, which is the excitation light source, the spectrometer and the 7-1 bifurcated fiber probe as shown in Fig. 1. For the excitation light source, we adopted LED (Innovation in Optics), with the excitation wavelength at 405 nm, as the LICF excitation source. The excitation source in principle can be either LED or laser-based. We have chosen LED for its low cost and long lifespan. The LED was driven in constant-current configuration using a laser diode driver circuit (Wavelength Electronics WLD33ND).

For the delivery of excitation light and collection of chlorophyll fluorescence, we used a 7-1 core bifurcated reflection fiber probe (Avantes, FCR-7UVIR400-2-ME). The core size of each fiber is 400 µm. At the distal end of the probe, the 405 nm LED light is delivered to the sample via the 7 fibers which are arranged in circular towards the peripheral of the probe, as illustrated in Fig. 1. The center fiber collects the chlorophyll fluorescence. The proximal end of the probe which house the 7 illumination fibers is connected to the LED source and the other end of the collection fiber is fed into the spectrometer.

A spectrometer (Avantes AvaSpec-ULS2048CL-EVO) was used for the acquisition of the chlorophyll fluorescence signal. The spectrometer came with a 2048-pixels linear array CMOS detector covering a spectral range from 200 to 1100 nm. The entrance of the spectrometer was fitted with a 50 µm slit which yields a spectral resolution of 2.5 nm. For sample acquisition, the integrating time was set at 0.5 s without averaging. The output optical power of LED measured from the distal end of the probe was approximately 15mW.

### LICF prototype for palm oil mill deployment

**Figure 2:**
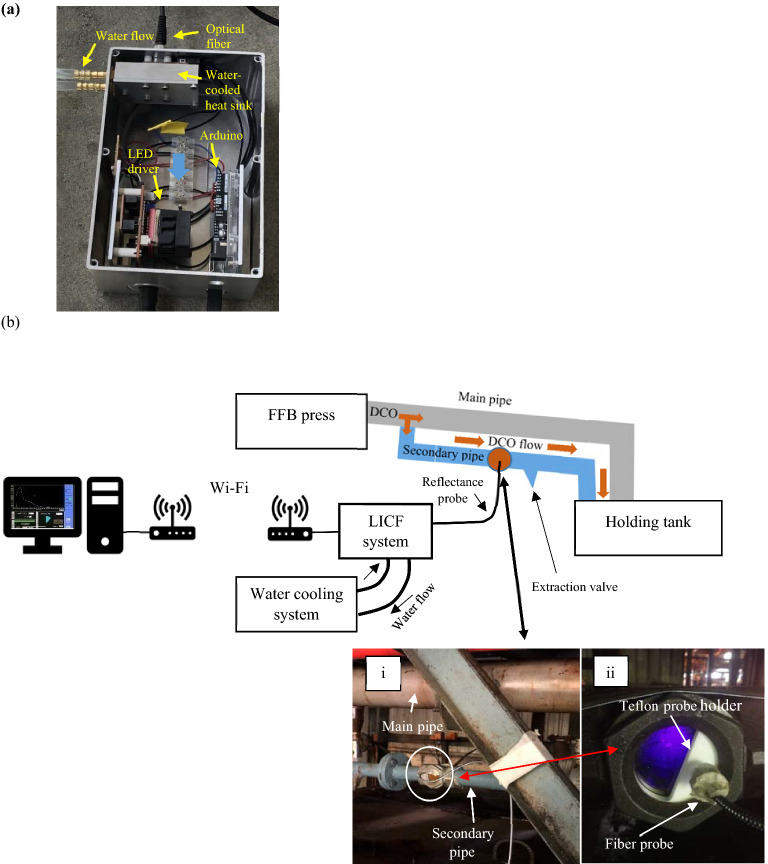
(**a**) LICF prototype light source system which include water-cooled heat-sink for keeping the LED light source from overheating, LED driver and Arduino board for collecting temperature sensor data. (**b**) Flow chart of oil palm FFB processing at palm oil mills and in-line Chlorophyll LICF prototype setup implemented at dilution & oil classification point, Bukit Kerayong oil mill, Malaysia. (i) The DCO, after pulp pressing, will flow through the main pipe to the holding tank. A secondary pipe is installed under the main pipe for the installation of the LICF probe. (ii) LICF probe being held by a teflon probe holder. The probe is separated from the DCO via a sapphire window. The telfon probe holder is designed to have an opening to enable visual inspection of DCO flow.

The LICF system prototype was developed and deployed in palm oil mill to test out its operation working in a harsh environment. The optical configuration is the same as that described in the above section. Unlike operating in laboratory, where the room is air-conditioned, the operating condition in the mill will be very different. Operating temperature in the oil mill is warm (between 33 and 40 °C) and this will pose a threat to the system especially the light source. To prevent thermal damage to the LED source, a water-cooled heatsink was specially designed and built. The photograph in Fig. 2a shows the LICF prototype light source system. The LED source was mounted in a water-cooled heat sink. Circulating water through the heatsink will help to prevent LED source from over-heating. Other than the LED source and the driver, there is also an Arduino board which connects to an array of temperature sensors to monitor temperature of the LED source, water and the environment.

Figure 2b illustrates schematic diagram of the LICF prototype installed at Bukit Kerayong oil mill, Sime Darby Plantation Berhad, Malaysia. LICF system is setup just after the FFB pressed to monitor the chlorophyll content in the DCO. To avoid disruption to mill operation, a secondary sampling pipe was fabricated and installed under the main pipeline. One end of the secondary pipe was connected to the main pipe and the other end terminates at the same storage tank where the main pipe terminates.

To probe the DCO sample, a flange was installed along the secondary pipe, in which the LICF fiber probe would be positioned, see Fig. 2(i). The flange was designed such that there was an opening on one side which was encased with a sapphire window (Edmund Optics), as shown in Fig. 2(ii). The sapphire window was selected for its good heat and chemical resistance properties. A teflon probe holder was then mounted at the outer side of the sapphire window. One half of the teflon probe holder will house the distal end of the LICF fiber probe. The other half of the probe holder was designed with an opening so that operator can monitor the DCO flow status during mill operation and to monitor the possible content build-up on the inner surface of the window. When that happens, the operator will have to remove and clean the window. As the probe is not in direct contact with the DCO, it will be easy for preventive maintenance on the fiber probe without the need to close the oil pipe valves and open the flange window. One of the advantages for using a fiber probe is so that the light source and spectrometer can be placed further away from hot pipeline. An extraction valve was added to the secondary oil pipe to facilitate sample collection for laboratory tests.

The LICF system communicates with the computer, located separately in an air-conditioned room via Wi-Fi connection. All the data was stored on the computer as well as in cloud. The measurements were recorded continuously at an integration time of 500 ms, averaging of 10 and time interval of 1 min between each recording. A total of approximately 1320 spectra will be collected per day.

### Statistical analysis

Principal component analysis (PCA) was used for this study to analyze the dataset collected fom LICF. It was carried out using Matlab (Mathworks, USA). The principal component loadings were applied to identify the variability in the dataset, highlighting the spectral components that differentiate FFB of different ripeness levels.

## Results and discussion

### Probing chlorophyll fluorescence response

**Figure 3:**
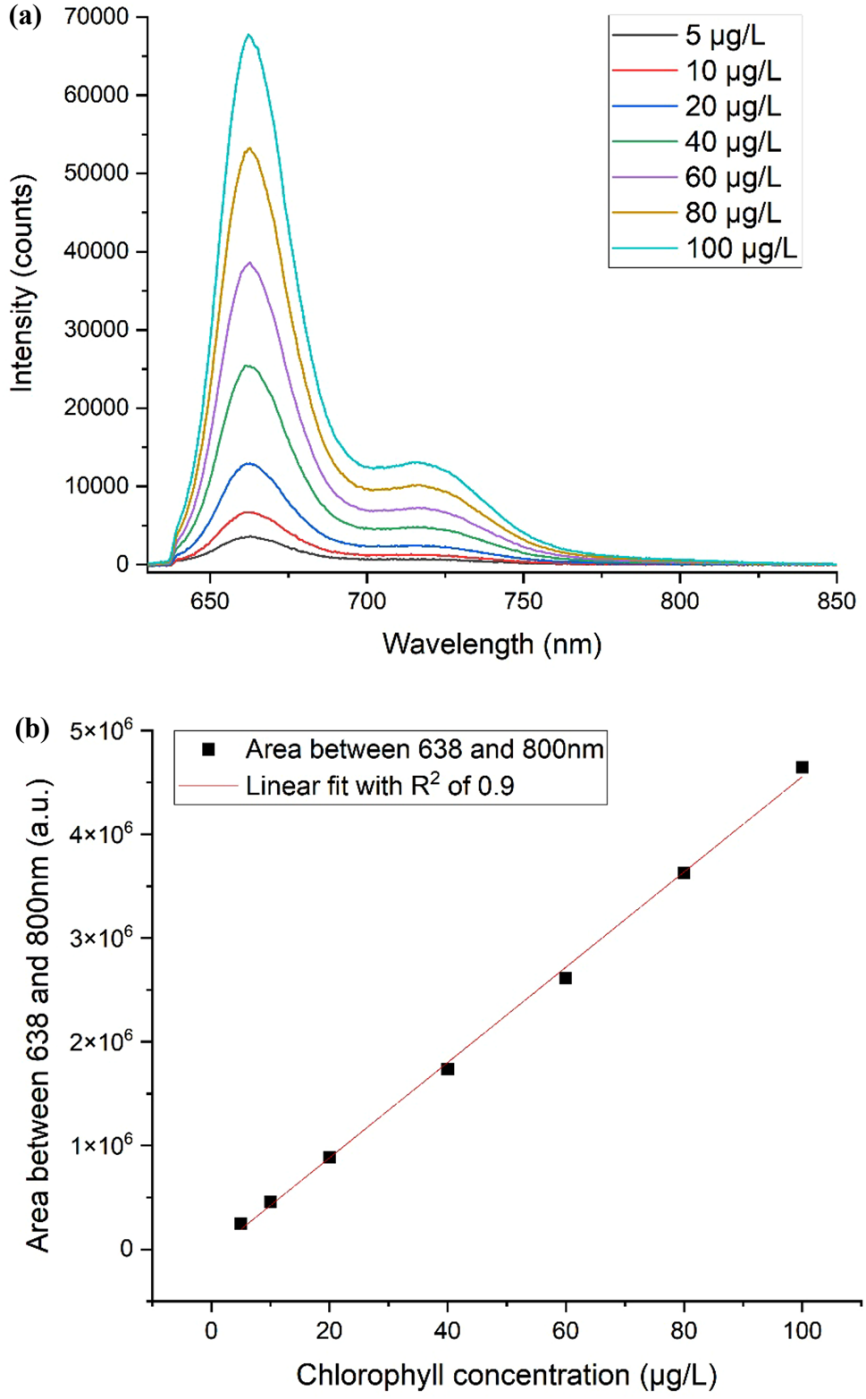
Shows the light-induced chlorophyll fluorescence spectra when excited with a 405 nm LED. (**a**) The LICF spectra of various chlorophyll concentrations in methanol. (**b**) The plot shows the linear relationship between chlorophyll fluorescence (fluorescence area) and chlorophyll concentration.

The LICF spectra of various chlorophyll concentrations in methanol are shown in Fig. 3a. The chlorophyll fluorescence signal exhibits peak at the region of 665 nm which is consistent with what is reported^33^. It is evident from the spectra that the chlorophyll fluorescence signal increases proportionally with chlorophyll concentrations. Figure 3b plots out the relationship between chlorophyll fluorescence area (from 638 to 800 nm) and concentration. The plot exhibits a linear relationship with a R-square of 0.9. These laboratory results demonstrate that LICF technique is sensitive and can be used for chlorophyll detection at a concentration of at least 5 µg/L.

### LICF response to chlorophyll content at different FFB maturities

In the paper by Sambanthamurthi et al.^21^, it was shown that the chlorophyll level in CPO is related to different stages of FFB maturities. As oil deposition in the mesocarp starts at about 15 WAP and continues until fruit maturity (20–22 WAP), palm oil from young fruits contains large amounts of chlorophyll and high moisture than oil from mature or ripe fruits^21^. Using the AOCS method, they reported that higher chlorophyll level in young FFB and it decreases when ripening progresses^20^.

**Figure 4:**
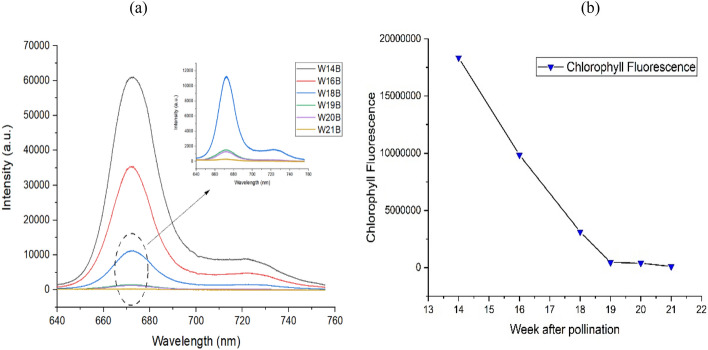
(**a**) LICF chlorophyll fluorescence spectra of CPO of FFB from different weeks after pollination. (**b**) Fluorescence area plot vs weeks after pollination.

In the following experiment, we explore the LICF approach to measure chlorophyll fluorescence level from oil that was extracted from FFB of different maturity stages. In cooperation with mill and palm estate managers, we prepared FFB at 14, 16, 18, 19, 20 and 21 WAP. The CPO from these batches of FFB was extracted and the chlorophyll fluorescence spectra were acquired via LICF lab setup. The results are plotted in Fig. 4. Figure 4a depicts the chlorophyll fluorescence signal at different WAP. The results show that chlorophyll fluorescence signal decreases sharply with increased palm fruits maturity from 14 to 19 WAP. This is in agreement with the observation made by Junkwon et al.^35^. The chlorophyll fluorescence signal, however, does not change much beyond 19 weeks, see Fig. 4b. This has been attributed to the present of immature fruits mesocarps containing large amounts of chlorophyll, which declined by about 17 WAP and accompanied by massive accumulation of carotenes as the fruit ripens from 19 to 21 WAP^19^. This is a promising result as it demonstrated the potential of LICF as a method to monitor the FFB maturity level in the mill.

### Correlating LICF to a lab measurement (AOCS) of oil mill samples

The aim of this section is to evaluate the LICF performance by referencing to the AOCS method. Measurements were carried out in the laboratory on DCO samples collected directly from the mill. Fifty-two DCO samples were obtained from Tennamaram KKS mill Malaysia over a period of 4 days. Each sample was collected at 30 min time interval from the start to the end of mill operation. Each sample was divided into two parts, one for the LICF measurement and the other for AOCS. The AOCS method serves as a reference for the LICF to benchmark against. Prior to measurement, each sample was first heated to temperature of above 60 °C to liquefy the content and then mixed well with a shaker.

**Figure 5:**
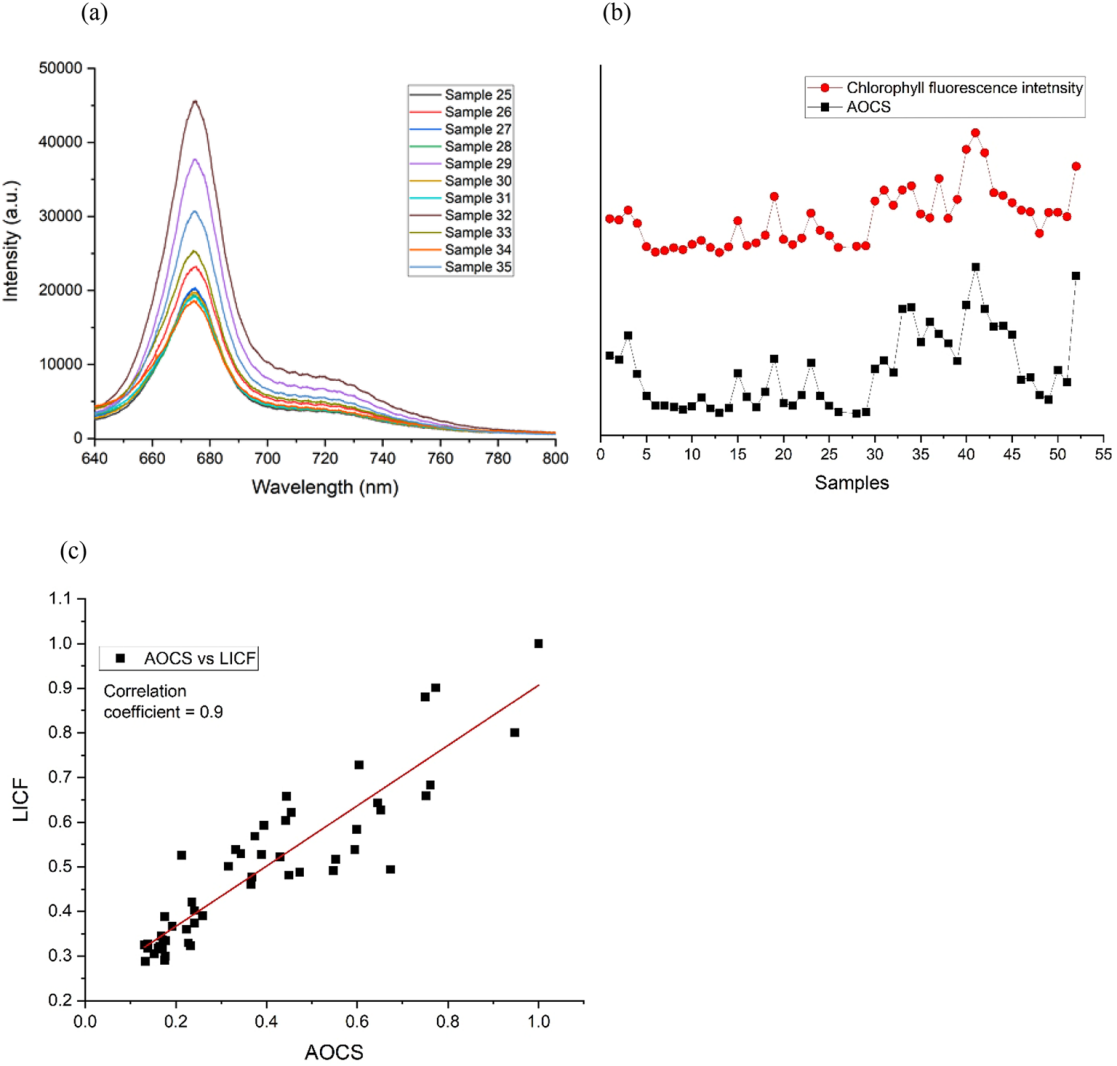
(**a**) Chlorophyll fluorescence spectra acquired from DCO samples collected from the mill. The plot shows 11 out of the 52 samples so that the graph is less cluttered. (**b**) A plot between LICF signal (red) and AOCS analysis (black) of 52 DCO samples. The graphs were normalized and offset for clarity. (**c**) Correlation plot between LICF and AOCS results yields a correlation coefficient of 0.9, suggesting that the LICF is a promising complementary method to AOCS.

Figure 5a shows the spectra plot of 11 chlorophyll fluorescence spectra for sample no. 25 to sample no. 35. The fluorescence spectra vary from samples to samples. Figure 5b shows a comparison plot between LICF (red) and AOCS (black) results. Each point along the LICF graph represents the area under the fluorescence curve between 640 and 760 nm. The LICF method achieved close correlation, correlation coefficient of ~ 0.9 (Fig. 5c), with the AOCS method. The promising result suggests that the LICF could be an alternative complementary approach to monitor LICF in the palm mill. In addition, the result also indicates that the chlorophyll fluorescence dominates and is less affected by fluorescence/artifacts signal from other debris within the DCO.

### Automated in-line LICF system in the palm mill operation

Being able to monitor chlorophyll level continuously provides mill operators valuable real-time information on the chlorophyll level. The LICF is recording the chlorophyll fluoresence continuous at an integration time of 0.5 s and averaging of 10. There was an time interval of 1 min between each recording.

**Figure 6:**
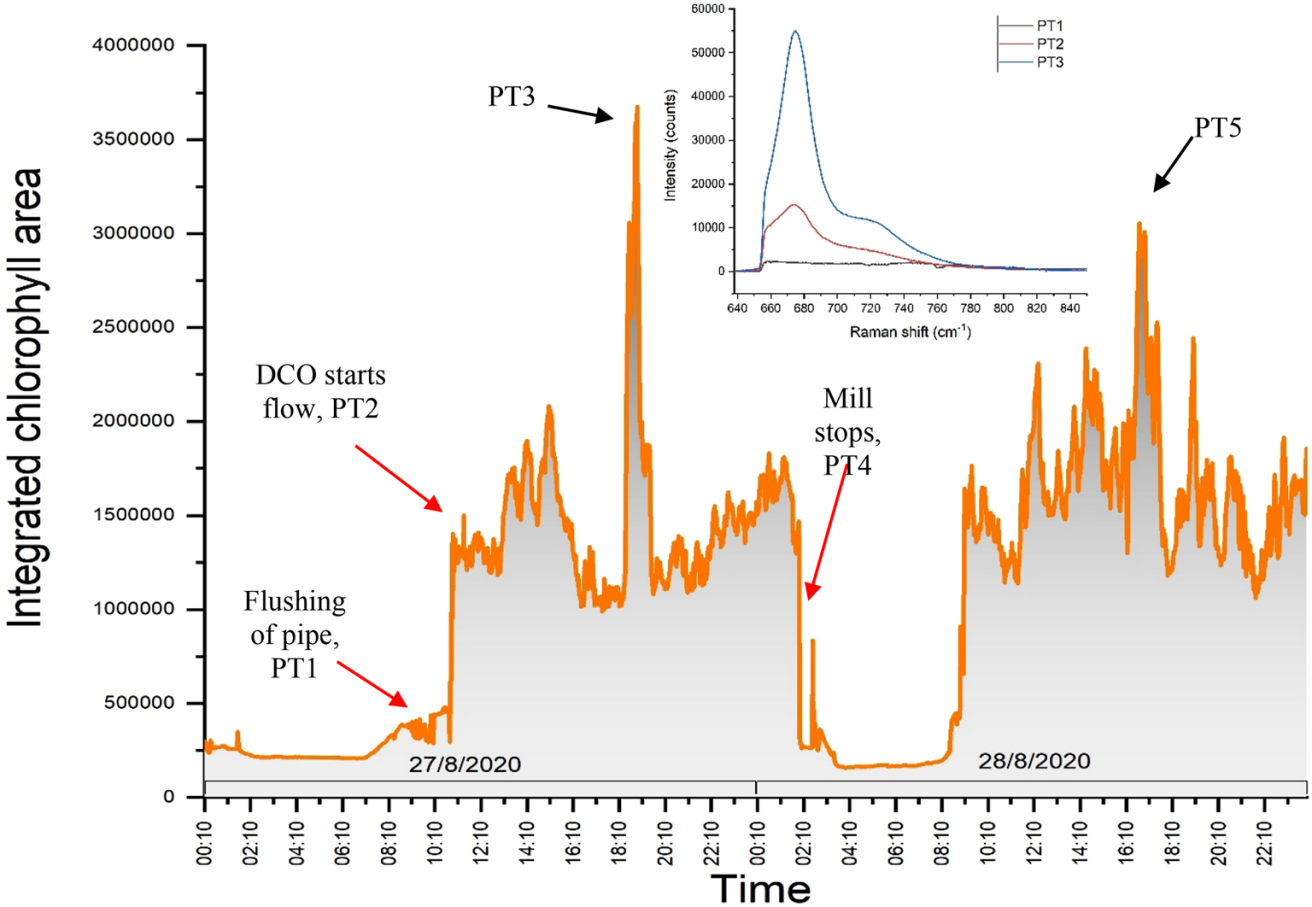
LICF operational plot for 2 days of mill run. PT1, PT2 and PT4 indicate the point in time of hot water pipeline flushing, DCO flows and end of mill operation, respectively. PT3 highlights the point when high chlorophyll level is being observed. Inset graph plots out the chlorophyll fluorescence at PT1, PT2 and PT3.

Figure 6 shows an example of LICF signal operational plot over the 2 day period, 27th and 28th August 2020. Each point on the graph represents area under the chlorophyll signal which spans from 650 to 850 nm. In additional to chlorophyll level information, the operational plot can also serve as a tool to monitor the mill operation. One of the important procedures, at the start of mill operation prior to palm oil extraction, is to flush the oil pipeline with hot water. Flushing of pipeline with hot water helps to clear any residues stuck inside the pipeline and the sapphire window. From Fig. 6, PT1 indicates hot water flushing of pipeline and PT2 highlights the point in time when DCO passes the pipeline. The latter indicates the start of oil production. The chlorophyll fluorescence varies throught out the mill operation as shown in the plot in Fig. 6. On the 27th August 2020, the chlorophyll fluoresence increases from 1210 to 1610 h. A spike increase in chlorophyll fluorescence level, PT3, was observed from 1810 to 2010 h. We observed similar chlorophyll spike on 28th August 2020, PT5. The high level of chlorophyll could be due to unripe FFB harvest.

**Figure 7:**
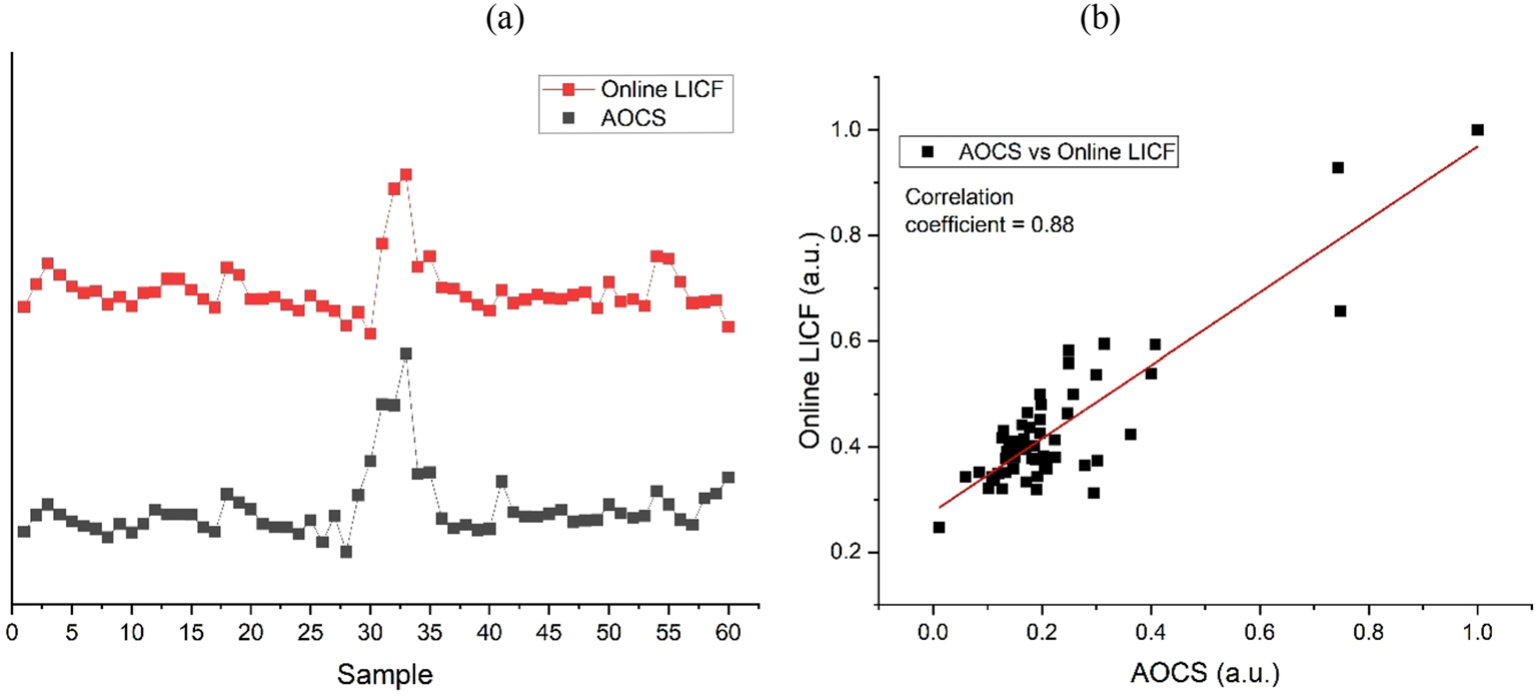
Comparison between the online LICF signal and the AOCS data. The LICF reading were recorded on the spot in the mill. (**a**) The DCO samples were also collected on the spot for AOCS measurement in the laboratory. (**b**) The correlation coefficient for between the two measurement is 0.88.

To examine the fidelity of the online LICF signal, we compare the LICF signal with the AOCS analysis result. A total of 60 DCO samples were collected and sent to the laboratory for AOCS measurement. The DCO samples were collect at 30 min time interval and each sample was collected via the extraction valve in sync with each LICF measurement. The AOCS analysis result along with LICF measurements are plotted in Fig. 7a. The LICF graph shows that sample 29 to sample 35 exhbited high chlorophyll fluorescence which infer high chloropyll content in the DCO. The result is in agreement with the AOCS data. The LICF method achieved a correlation coefficient of 0.88 with the AOCS analysis as shown in Fig. 7b. This is close to what was had been obtained from laboratory experiment which yielded a correlation coefficient of ~ 0.9.

### FFB ripeness assessment using LICF

Palm oil yield and oil extraction rate (OER) is related to the FFB ripeness. The oil yield and OER is high for ripe FFB and low for unripe FFB^36^. As the level of chlorophyll in the FFB is related to its ripeness, we could use the LICF method to monitor the ripeness of the FFB that were delivered to the mill. Together with the assistance from mill management and palm oil plantation teams, we conducted a controlled experiment with LICF system to monitor FFB ripeness level. In this experiment, we segregated the FFB into 3 categories, namely 0% unripe, 5% unripe and 10% unripe FFB brunches. Level of ripeness is gauged by counting the number of empty sockets on each fruit for 100 number of FFB in the pile of FFB. The FFB was processed in the mill according to the category and chlorophyll signal was collected through the pipeline. A total of 645 spectra, from all 3 levels of ripeness, were collected and tabulated into a single dataset.

**Figure 8:**
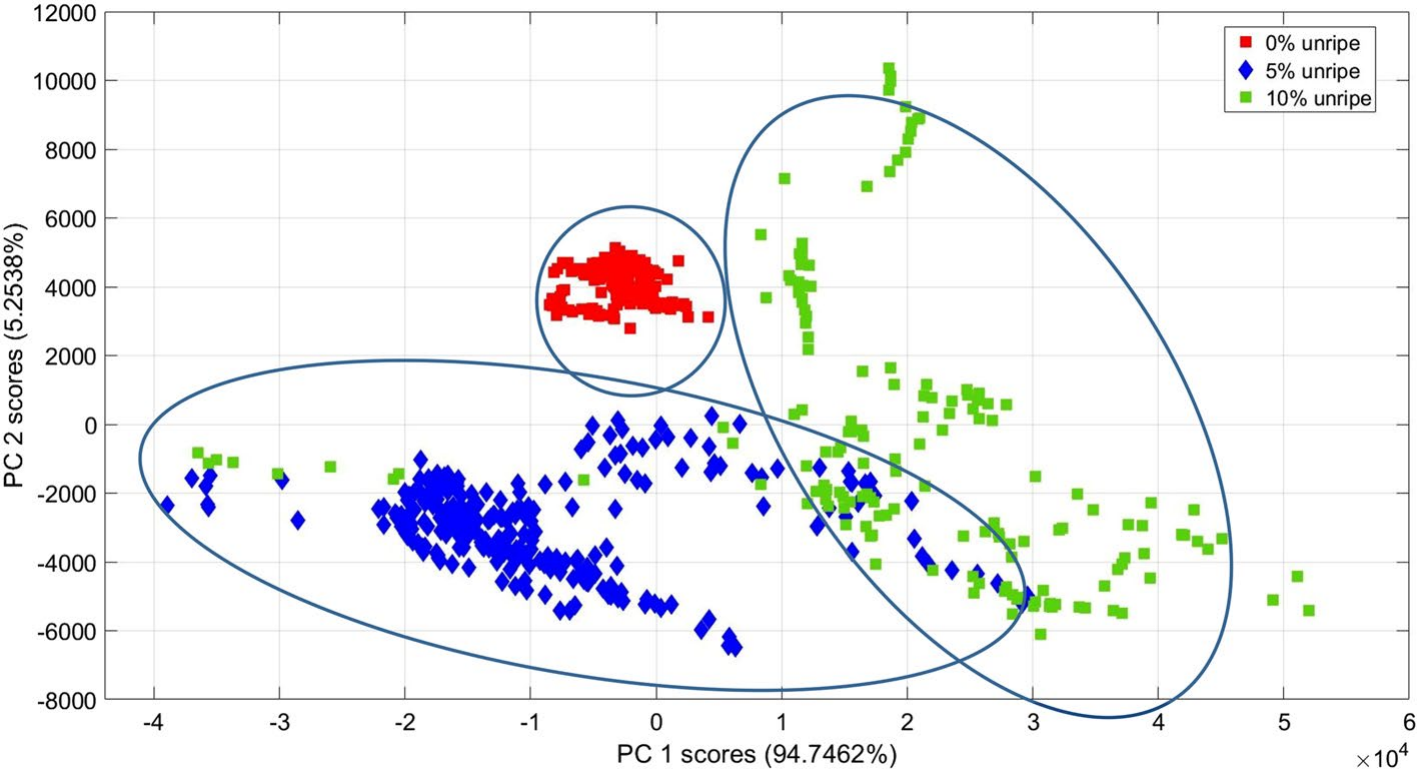
PCA scattered plot between PC1 and PC2 of chlorophyll fluorescence spectra from DCO extracted from 0% unripe (red), 5% unripe (blue) and 10% unripe (green) FFB.

**Figure 9:**
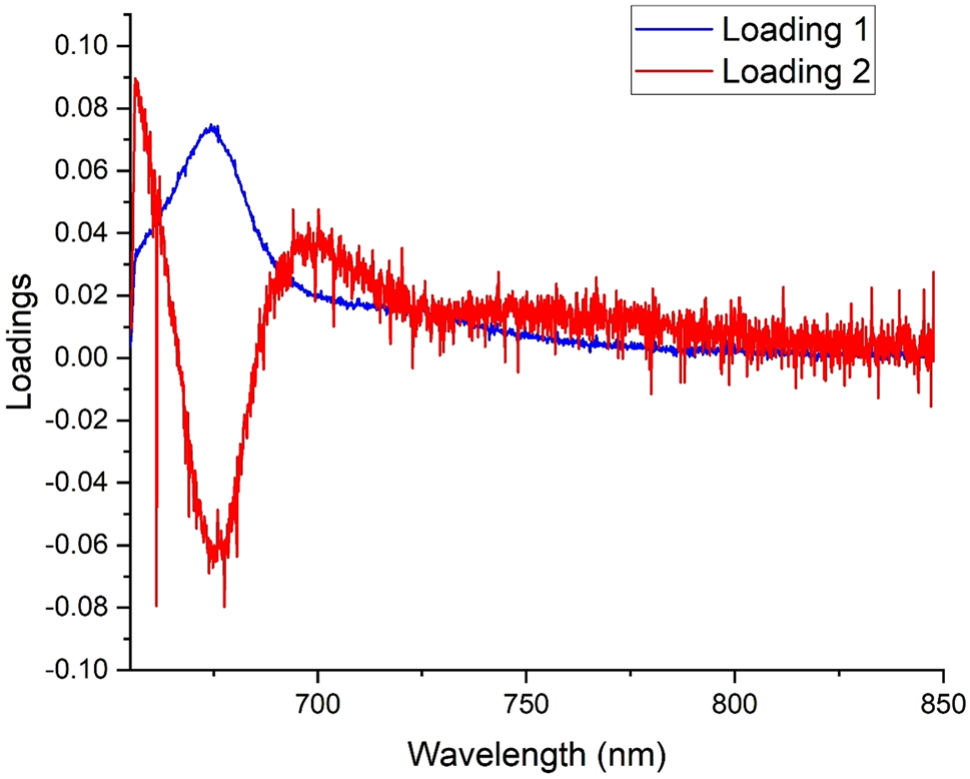
PC1 and PC2 loading plots of 0%, 5% and 10% unripe FFB samples.

Figure 8 illustrates the principal component (PC) scores plot for 0% unripe FFB (red), 5% unripe FFB (blue) and 10% unripe (green) FFB. Here the LICF spectra from FFB with different level of unripeness can be distingushed from each other based on the level of chlorophyll fluorescence. The first two PCs for this dataset address all of the net variance in the spectral dataset. From Fig. 8, all the sample from the 0% unripe FFB cluster tightly together and well-separated from the rest of the samples. This can be explained by the extra low chlorophyll level coming from the 0% unripe FFB samples (red). The LICF spectra from 5% and 10% unripe FFB are also separated from each other with a small portion of overlap. Here the LICF spectra from FFB with different level of unripeness can be distingushed from each other based on the level of chlorophyll fluorescence. The separation of the 10% unripe FFB from the group of 0% and 5% unripe FFB is due to the higher level of chlorophyll fluorescence, as shown in the positive loading 1 plot (blue) in Fig. 9. The separation between 0% unripe and 5% unripe FFB is shown in Fig. 9 the negative loading 2 plot (red), which is due mainly to the higher level of chlorophyll fluorescence from the 5% unripe FFB.

**Figure 10:**
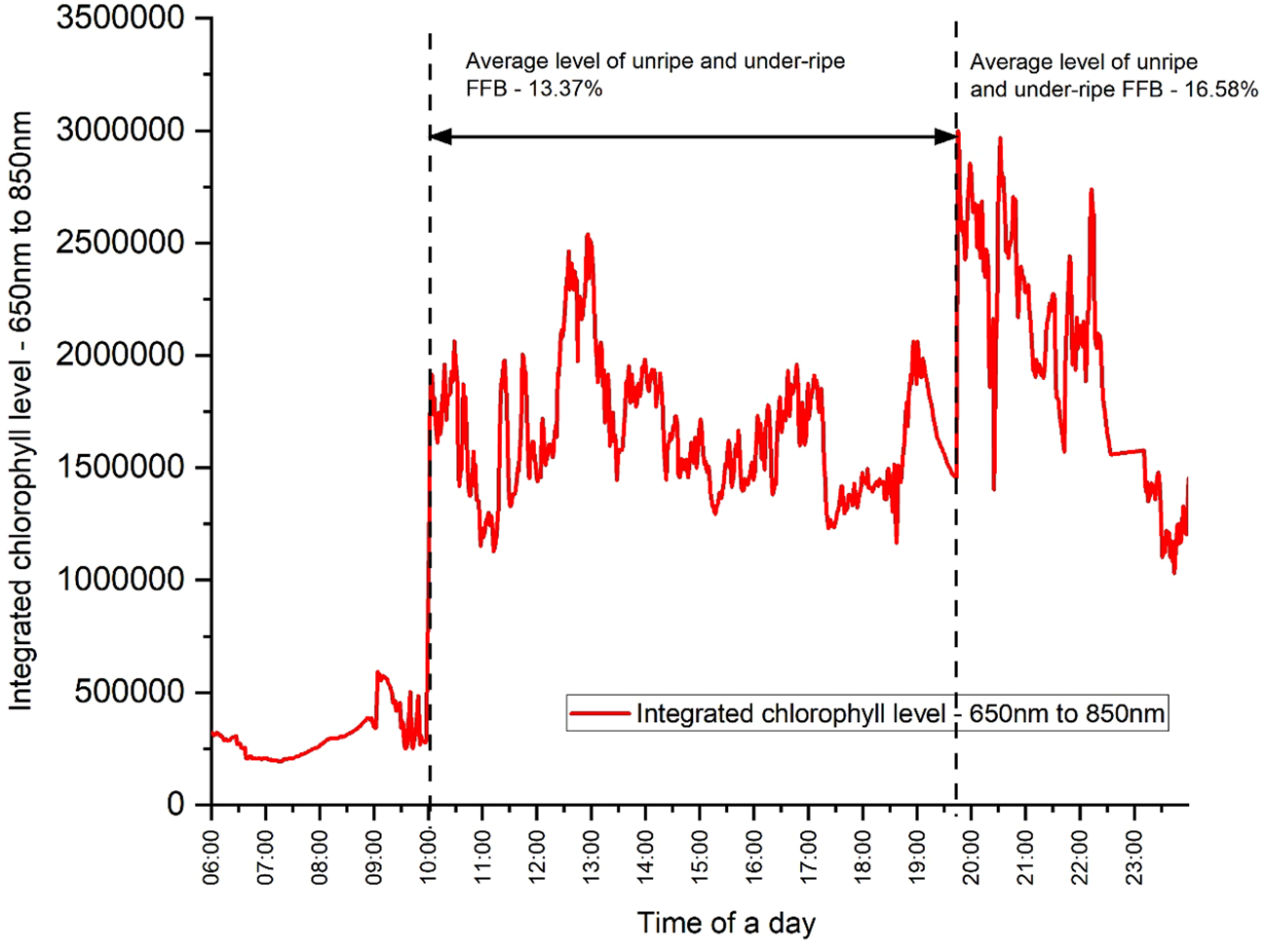
A single day LICF data and the corresponding level of unripe FFB provided by the ripeness graders.

Pushing forward, we compared the chlorophyll level for a normal day’s operation with the FFB ripeness in according to the usual ripeness assessment done by the mill experts. Figure 10 shows the unsupervised LICF data for a whole day of mill operation. From the plot, we noticed a higher chlorophyll signal between 19:50 h and 23:59 h. According to the mill operators, there was a switched to a different batch of FFB that was received in that morning. Validating with the mill FFB ripeness grader team revealed that the morning batch of FFB has an unripe and under-ripe FFB level of 16.58%, whereas the afternoon FFB batch, which was received in the previous day, has an unripe and under-ripe FFB level of 13.37%. This means that in this particular production run the FFB received in the very morning had a higher level of unripe FFB. The LICF data agrees well with the ripeness assessment data. This promising result validates the system potential capability for indirect real-time assessment of FFB ripeness based on chlorophyll.

## Conclusions

In this study, we have developed and evaluated the LICF prototype for fast real-time chlorophyll monitoring in DCO samples in the mill. Despite the complex mixture content of the DCO, we have demonstrated that we are able to obtain good chlorophyll correlation data both in the laboratory and in live mill settings. A correlation between real-time LICF signal and off-line AOCS method yields a correlation coefficient of ~ 0.88. The data generated from the spectra produced by the LICF system can differentiate between different FFB ripeness by analyzing the degree of chlorophyll fluorescence. The promising results demonstrate the feasibility of deploying LICF for real-time chlorophyll monitoring of palm oil during the mill production.

## Data Availability

The datasets used and/or analysed during the current study available from the corresponding author on reasonable request.
